# Adult diet in England: Where is more support needed to achieve dietary recommendations?

**DOI:** 10.1371/journal.pone.0252877

**Published:** 2021-06-23

**Authors:** Dianna M. Smith, Christina Vogel, Monique Campbell, Nisreen Alwan, Graham Moon

**Affiliations:** 1 Geography & Environmental Science, University of Southampton, Southampton, United Kingdom; 2 NIHR Applied Research Collaboration (ARC) Wessex, Southampton, United Kingdom; 3 MRC Lifecourse Epidemiology Unit, University of Southampton, Southampton, United Kingdom; 4 NIHR Southampton Biomedical Research Centre, University of Southampton and University Hospital Southampton NHS Foundation Trust, Southampton, United Kingdom; 5 School of Primary Care, Population Sciences and Medical Education, University of Southampton, Southampton, United Kingdom; Xiamen University, CHINA

## Abstract

**Background:**

Small-area estimation models are regularly commissioned by public health bodies to identify areas of greater inequality and target areas for intervention in a range of behaviours and outcomes. Such local modelling has not been completed for diet consumption in England despite diet being an important predictor of health status. The study sets out whether aspects of adult diet can be modelled from previously collected data to define and evaluate area-level interventions to address obesity and ill-health.

**Methods:**

Adults aged 16 years and over living in England. Consumption of fruit, vegetables, and sugar-sweetened beverages (SSB) are modelled using small-area estimation methods in English neighbourhoods (Middle Super Output Areas [MSOA]) to identify areas where reported portions are significantly different from recommended levels of consumption. The selected aspects of diet are modelled from respondents in the National Diet and Nutrition Survey using pooled data from 2008–2016.

**Results:**

Estimates indicate that the average prevalence of adults consuming less than one portion of fruit, vegetables or 100% juice each day by MSOA is 6.9% (range of 4.3 to 14.7%, SE 0.06) and the average prevalence of drinking more than 330ml/day of SSB is 11.5% (range of 5.7 to 30.5%, SE 0.03). Credible intervals around the estimates are wider for SSB consumption. The results identify areas including regions in London, urban areas in the North of England and the South coast which may be prioritised for targeted interventions to support reduced consumption of SSB and/or an increase in portions of fruit and vegetables.

**Conclusion:**

These estimates provide valuable information at a finer spatial scale than is presently feasible, allowing for within-country and locality prioritisation of resources to improve diet. Local, targeted interventions to improve fruit and vegetable consumption such as subsidies or voucher schemes should be considered where consumption of these foods is predicted to be low.

## Introduction

Promoting a healthy diet rich in fruit and vegetables is one of the most widespread public health campaigns globally, with five portions a day or at least 400 grams of fruits and vegetables (commonly referred to as 5-A-Day) recommended in the UK and most other high-income countries [1]. Although the UK’s Eatwell Guide emphasises and strongly recommends adherence to the 5-A-Day target, these guidelines also state that foods high in free sugars (inclusive of sugar sweetened beverages-SSBs) should not exceed five percent of total daily energy intake [2]. The latter recommendation is based on the strong association between SSB consumption and weight gain, particularly in adult women [3], and the higher consumption of SSB amongst younger adults compared to older populations [4]. A substantial body of literature has identified poor health outcomes, including obesity and type two diabetes, associated with low consumption of fruits and vegetables and high levels of sugar, especially sugar sweetened beverage (SSB) consumption [5–7].

Despite considerable research showing the health benefits of greater fruit and vegetable consumption [8], only 30% of the UK population [9] and 26% of the English population [10] achieve the 5-A-Day target. The UK adult population is also said to consume almost three times the recommended amount of free sugars, with sugar sweetened beverages (SSBs) such as soft/fizzy drinks accounting for a large proportion of the daily sugar intake of both adults and children [11]. Among lower income groups the consumption of produce is considerably below recommendations and SSB intake above recommendations which is likely contributor to their poorer health outcomes [12]. In addition, recent studies have found obesity and type 2 diabetes to be two of the most prominent risk factors associated with COVID-19. Diet, especially those low in fruits and vegetables and high in saturated fat, refined carbohydrates and sugar (typically referred to as a Western dietary pattern) is identified as one of the main drivers of this risk [13]. These findings which when coupled with sub-optimal diets in the UK, help to explain the Government’s urgency in tackling the diet and obesity “time bomb” through the recently announced “Better Health” campaign [14].

The aim of this paper is to provide small-area data on population diet outcomes to inform public health policy in England. The research presented here demonstrates the utility of devising local-level estimates of less desirable diet behaviours to enable public health practitioners to target interventions effectively and support improvements in population dietary behaviour. Over time this should have a positive impact on population health by reducing diet-related health inequalities. This modelling process will allow government, national and local, and civil society to quickly identify priority areas for appropriate interventions and may be replicated in any setting where reliable diet surveys and population censuses are available. Small-area estimation methods have been used previously to model health behaviours and outcomes in local areas, including obesity [15, 16], mental health [17], smoking [18], diabetes [19–21] and alcohol consumption [22]. Outputs from these models are used to inform planning for healthcare service provision within local governments. Modelled data on health outcomes, where no small-area measures exist, are publicly available on government websites to support local planning and prioritisation of funding to address health inequalities by national government (See for example localhealth.org.uk) [23].

Though models have been developed to explore the impact of a sugar tax on population consumption, to date there are no efforts to model local-level consumption and predict which areas may see the biggest reduction in consumption following the 2018 sugar tax. Now that public health is ultimately the responsibility of local authorities underneath the Health and Social Care Act of 2012 [24], such knowledge empowers local public health strategy. In addition, consideration could be given to more effective targeting of national voucher schemes such as Healthy Start, which provide funds to purchase fresh or frozen fruit and vegetables to lower income households, [25] or extending local interventions including Rose vouchers which give an additional £3/week/child on top of Healthy Start vouchers [26]. Further modelling of diet following modifications to the provision of vouchers, perhaps by increasing the voucher value as inflation impacts on food prices, could provide an indication of the anticipated impact on population health [27]. For such scenario modelling to be feasible, local-level data or reliable estimates must be available.

To address health inequalities, interventions must be considered to support optimal diets for all people. Dietary quality is one area where health policy has the scope to influence substantial changes to overall diet patterns in the population [28]. Few studies have explored the potential impacts of higher fruit and vegetable consumption on population health specifically, however one recommended policy intervention for increasing these elements of diet is subsidising fruit and vegetables [28]. In June 2020 the House of Lords published recommendations to increase the value of Healthy Start vouchers as part of plans to support a diet high in fruit and vegetables [29]. Alongside SSB consumption, it would be beneficial to understand how consumption of low levels of fruit and vegetables vary geographically in the adult population, to consider appropriate interventions to improve dietary behaviours in areas where there is the greatest potential impact.

There is evidence that price increases may reduce consumption of less healthy items, with sugar often a priority area for these interventions. The introduction of a tax on SSB in parts of the US reduced population-level consumption with small levels of taxation [30], however, a recent systematic review concludes that price increases need to be at least 20% to achieve a reduction in consumption [28]. Public health campaigns on reducing sugar consumption have put pressure on the government to enforce reformulation of food products to reduce sugar intake in the UK and led to the creation of a ‘sugar tax’ implemented in 2018 [31]. This tax affects food and drink with high levels of free sugars, with one key category of foods including SSB. If the SSB producers do not reformulate products to reduce the amount of sugar, then a tax is applied to the producers with a higher tax on drinks with 8 grams or more per 100ml and a lower level of tax on drinks with 5 grams or more per 100ml [32]. The sugar tax has resulted in some reformulation.

Evaluations of taxation policy to promote healthier dietary choices recommend a combination of increasing the cost of less healthy options and subsidising healthy items including fruit and vegetables [33]. Large-scale models of populations have been used to estimate the cost implications of diet change [34], public health impact of taxation on consumption of fast food in the US [35] and SSB in English Local Authority Districts [36]; here we extend the modelling approach to examine smaller areas and identify neighbourhoods where targeted interventions are most needed to reduce inequalities in population-level health outcomes related to diet.

To address the aims of modelling diet in local areas models of four diet variables are completed: i) daily fruit intake, ii) daily vegetable intake, iii) daily fruit & vegetable, and iv) daily SSB intake. Nationally representative data of adult diet (≥16 years) from the National Diet and Nutrition Survey were used to model at the neighbourhood level, specifically the Middle Super Output Area (MSOA, n = 6791) level which approximates a large neighbourhood with an average population of 7000 and sits within Local and Unitary Authorities. This creates data which may provide estimated baseline consumption to later evaluate implemented interventions where local data are not collected. The estimates will also aid local policy decision-making such as prioritising areas for healthy food subsidies or planning restrictions for unhealthy food outlets.

## Materials and methods

The development of small-area estimates requires three stages. First, potential predictor variables are identified and assessed for their utility in predicting the target outcome or behaviour using correlation and regression analyses. Then the health outcomes or behaviours are estimated using the most appropriate predictor variables by applying small-area estimation methods (here, spatial microsimulation). Finally, measures of validity and uncertainty for the estimates are developed using the single level regression outputs from the first stage and new multilevel regression models.

Dietary data were collated from the National Diet and Nutrition Survey (NDNS) datasets from 2008–2016 [37]. These data were accessed from the UK Data Archive (https://www.ukdataservice.ac.uk/) and are not available for sharing as per our end user agreement. Ethical approval for this project was granted by the University of Southampton ethics committee (reference 28610). As this study used secondary data from a national survey, the ethics committee deemed it appropriate use of the data and further consent was neither feasible nor required. The NDNS is the foremost diet survey in the UK which captures data on all food and drink consumed over a four-day period using a food diary, providing a reliable measure of diet quality compared to diet screeners included in less-specific health surveys [38]. In the NDNS the pooling of eight years of data provides a larger sample size for the modelled estimates. This annual cross-sectional survey samples individuals aged 1.5 years and over living in private households, with a target of 1000 respondents each year.

In the NDNS, an (unweighted) food diary dietary assessment method was used to capture detailed information on all foods and beverages actually consumed by participants, over a four-day period. Participants were trained to complete food diaries and estimated portion sizes using generic household measures (e.g. two thick slices of bread or four tablespoons of peas) or weights from labels (e.g. 420-gram tin of baked beans or 330 ml can of lemonade). Diaries included pictures of ten of the most frequently consumed foods, which made it easier for individuals to describe portion sizes in a user-friendly manner and helped to further reduce participant burden. Survey interviewers made follow-up checks (in person or via telephone) on the second or third day of recording to review diaries, check for and fill in missing details, provide guidance on how to improve recording and motivate participants to continue recording. In keeping with the Eatwell Guide, the survey defined one portion of fruit/vegetable as 80 grams and the total achievement of the “5-A-Day” national fruit and vegetable target as the consumption of at least five portions or 400 grams of fruits and vegetables daily. Unsweetened, 100% fruit juice and smoothies were included in fruit portion calculations and counted as one portion but were not to exceed 150 millilitres daily. Therefore, consumption of, for example, 300 millilitres of fruit juice in a day would still only be counted as one portion (150 millilitres) towards the overall 5-A-Day target. One portion of dried fruit was 30 grams, which was the equivalent of 80 grams of fresh fruit. Beans and pulses (e.g. lentils, kidney beans) counted towards one portion of vegetables per day, regardless of the amount consumed in a day. Data on portions of fruit and vegetables consumed included components of prepared dishes as well as pieces of fruit or vegetables whether fresh, frozen or canned [37]. We modelled the consumption of SSB, portions of fruit, portions of vegetables and the total portions of fruit, vegetables and 100% fruit juice (up to 150 ml) separately. Portions of SSB were recorded in millilitres (ml) per day. Diet outcomes were modelled for those age 16 years and above to correspond with health outcomes measured in national surveys including the Health Survey for England [10].

### Selection of appropriate predictor variables

In order to have accurate models of the diet outcome of interest, appropriate predictor variables need to be identified that are present in both the national survey used to develop the small-area estimates and in the population dataset such as the Census. Preliminary statistical analysis is conducted to identify variables which meet these criteria, and are also evident in the academic literature as predicting the behaviours [39]. The sociodemographic variables most strongly associated with consumption of each of the four dietary variables were identified through chi-square correlation analysis then logistic regression. The sociodemographic variables tested included age, sex, ethnicity, National Statistics Socioeconomic Classification (NSSEC), marital status, housing tenure and educational qualifications. These variables were considered for two reasons. First, they are associated with diet quality in individuals in other datasets and research [11]. Second, they are all also collected as part of the decennial Census in the UK, which is required for the method of small-area estimation used here. Variables were considered highly correlated if p < 0.001. The coding of each variable was specified as follows: SSB were coded at none, up to 330ml (reflecting one can of fizzy drink) of SSB and over 330ml; portions of fruit, vegetables and combined fruit, vegetables and 100% fruit juice consumed are coded as 0–0.99, 1–4.99, 5+ portions per day. Each portion is based on an 80g portion size for fresh fruit or vegetables or 30g of dried produce.

Binary logistic regression models for four outcomes (< 1 portion fruit, <1 portion vegetables, < 1 portion fruit, vegetable and juice or >330 ml SSB) was the next stage of selecting appropriate predictor variables for these relatively poor diet behaviours, with the final step the creation of multivariate logistic regression models for each the four diet outcomes to identify the optimal combination of predictor variables in each instance. The results from the multivariate logistic regression model were also used in the creation of credible intervals around the estimated prevalence of diet behaviours, as described later in this section [40]. The scale of these estimates, MSOAs, are similar in size to the primary sampling units used to select areas and populations to sample for the national surveys. This MSOA scale is the level used in other small-area estimates created by the Office for National Statistics [41]. MSOAs often nest within wards, enhancing the utility of the estimates for public health strategists who use ward-level data.

### Creating local estimates of diet: Small-area estimation

The method of creating the local diet behaviour estimates is one approach to small-area estimation, spatial microsimulation. The spatial microsimulation algorithm used is based on iterative proportional fitting (IPF) with full details of the method provided elsewhere [39]. This method has been used successfully in England to estimate common mental disorders and alcohol consumption [17] in England and smoking in New Zealand [42]. IPF approaches have been applied successfully in England to estimate obesity [15] and smoking [18] and to estimate a range of health outcomes in Scotland [43]. The local population data for MSOAs was based on the age-sex mid-year population estimates produced by the Office for National Statistics (ONS) for 2018 [44] and the 2011 Census data [45]. Individual socioeconomic classification was identified using the National Statistics Socioeconomic Classification (NSSEC) scheme [46]. Data on NSSEC, ethnicity, marital status, housing tenure and educational qualifications were scaled to reflect the 2018 age-sex population estimates. Ethnicity was coded into four categories as White, Asian, Black and Other or Mixed. NSSEC data were collapsed into four categories of socioeconomic status based on the household representative person’s categorisation of employment (High, Moderate, Low, Unclassified [including unemployed or unclassified]) from the original eight, following guidance from ONS [46]. Marital status was coded as single, married/civil partner, divorced/widowed/other. Housing tenure is coded as owned, social rented, private rented. Finally, educational qualifications are recoded to degree or above, below degree level/other, no qualifications, in full time education.

**Fig 1:**
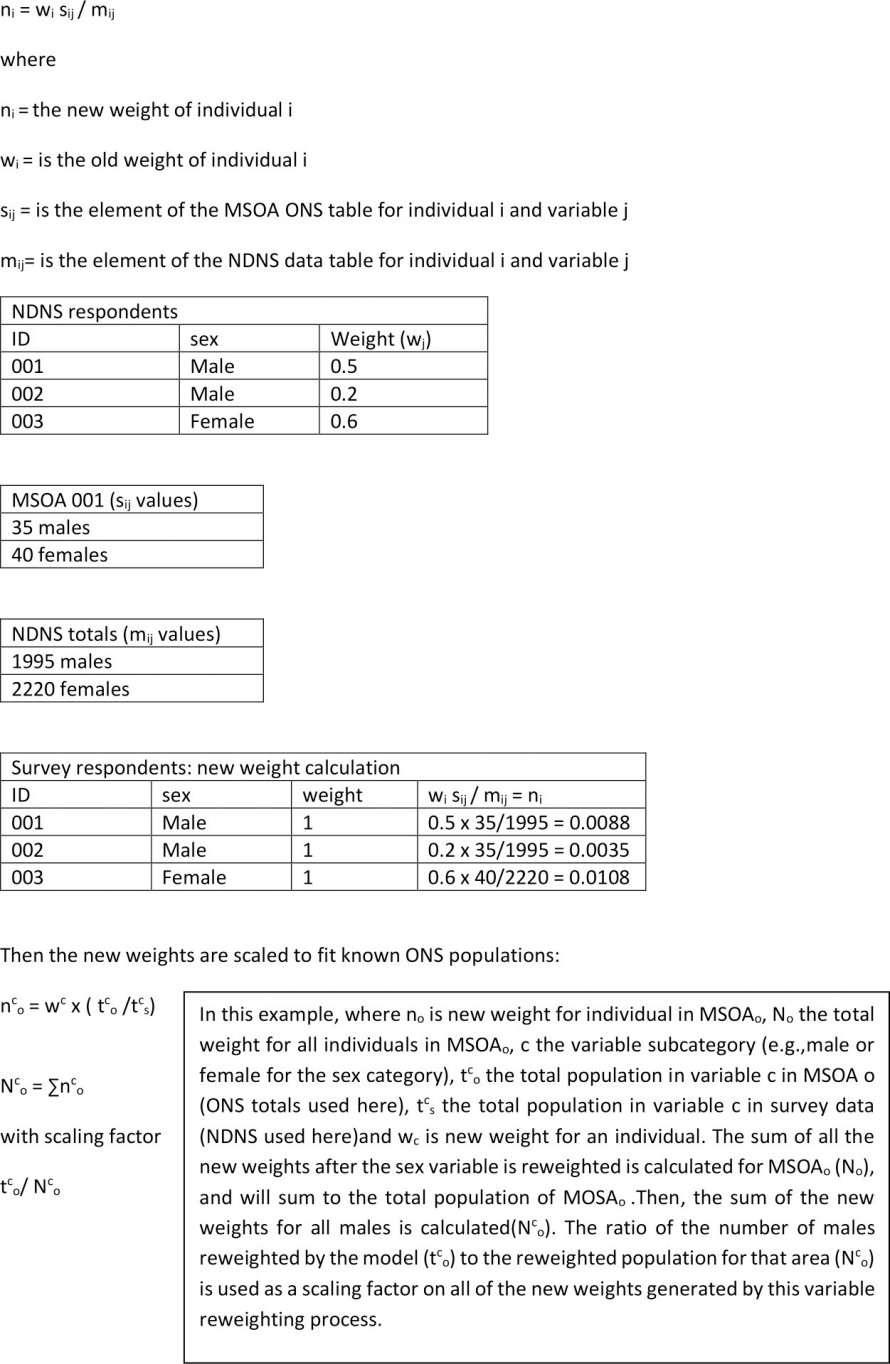
Worked example of IPF routine for SAE.

The IPF approach calculates the probability of each eligible respondent from NDNS (complete case analysis, those who provided all necessary diet and demographic data) to live in each MSOA across England. The probability is calculated by matching respondents from NDNS to known residents in the Census based on the demographic variables which were shown to best predict the selected dietary behaviours from the correlation analysis. The probabilities for each person to live in each area are then summed to total the local population (based on the ONS 2018 data), and the associated dietary behaviours are attached to these new area populations. The result of the summed probabilities provides an estimated prevalence for each diet behaviour in each MSOA. Fig 1 shows a worked example of the reweighting and subsequent rescaling of the new probabilities to fit ONS population data. For this paper, this process was repeated four times, once for each dietary variable.

### Identifying priority areas for interventions

Following the creation of local-level prevalence estimates of diet outcomes, potential priority areas for interventions were identified. This was done by selecting the top decile of poorer diet behaviour (prevalence) for two key measures, SSB consumption of >330ml/day and people consuming less than one portion a day of fruit and vegetables. The decile distribution is a common approach in public health prioritisation [47] as data are not often normally distributed so not well suited to statistical tests. To confirm that the top deciles differed significantly from other MSOAs a Mann-Whitney U test was completed. To aid national prioritisation, a test of spatial autocorrelation of MSOAs where there is high prevalence of these two outcomes was completed using Local Moran’s I. This method identified spatial clusters of MSOAs where the diet outcome is more common and highlighted local authorities where interventions could be focused. We applied the Local Moran’s I models to the point (prevalence) estimates of high SSB consumption (the percentage of the adult population consuming more than 330 ml of SSB per day) and low fruit and vegetable consumption (the percentage of the adult population consuming less than one portion of fruit and vegetables in a day). The cluster analysis used the Inverse Distance spatial conceptualisation model and we did not specify a minimum distance for spatial neighbours [48].

### Assessing validity of small-area estimates: Internal validation and credible intervals

To provide validation of model estimates, previous researchers have aggregated estimates to a higher-level geography where the prevalence of an outcome or behaviours is known [17] or compared estimates to related outcomes, such as the prevalence of diabetic amputations to validate diabetes prevalence estimates [21]. Earlier models of SSB consumption in England ran a series of sensitivity analyses to provide more confidence in estimates [36]. Previously this method of small-area estimation for smoking prevalence has been validated using data from a full population census in New Zealand [42]. Another consideration is the internal goodness-of-fit in the modelled populations, with the preference being standardised Total Absolute Error (TAE) between the observed and estimated demographic variables used to predict the outcomes (here, age, sex, and so on) [49]. Observed values are gained from the Census and ONS and the mean TAE for each constraint variable is calculated across the 6791 MSOAs in England. As there are no readily available comparable datasets of SSB or fruit and vegetable consumption in England at the MSOA level [38], the focus of model fit is on the internal comparisons.

Credible intervals for the estimated prevalence of diet behaviours were calculated using the outputs from multiple regression models for each of the diet outcomes and using a multilevel modelling framework. Details of this process are explained fully in Whitworth et al. 2017 [40]. Briefly, the data from NDNS has already been analysed in single-level multiple regression models for each diet outcome. The associated sampling data from NDNS for each respondent (sample cluster, region) is used to build a multilevel model for each outcome where individuals are nested within the sample clusters, nested within regions. The outputs from these multilevel models indicate the variance at level two (sample clusters). This captures the uncertainty present at the cluster level, units that are of a similar size to an MSOA. The variance is replicated stochastically to represent a range of 1000 possible values for each MSOA around the point estimate derived from small-area estimation, and the upper and lower credible intervals are taken from the 2.5^th^ and 97.5^th^ percentiles of this distribution. The inclusion of credible intervals around the local estimates is a valuable addition to the small-area estimates of diet as most previous research using this method of small-area estimation does not include measures of uncertainty around the point estimates [40].

All statistical analysis was completed using SPSS v 25 except for the credible interval multilevel models which were calibrated using MLwiN (3.01). The spatial microsimulation model is written in R code and the maps were created using ArcGISPro 2.7.

## Results

The estimated patterns of SSB, fruit, vegetables and the portions of fruit and vegetables, including 100% juice, consumed each day from the NDNS datasets are summarised in Table 1 along with respondent demographics. Here, the predictor variables that were most significant following the binary and multivariate logistic regression analysis are retained (sex, age, marital status, housing tenure and educational qualifications) while those which were less significant were dropped from the analysis (NSSEC, ethnicity). Tenure and educational qualifications were highly correlated so were not used in the same models.

**Table 1:** Summary of respondents and crosstabulations with outcome variables.

	SSB consumption		n = 5160				Total portions (F&V)		n = 5150			Fruit portions	n = 5157				Veg portions	n = 5160		
	no SSB	1-329ml	330ml +	p value		< 1 portion	1–4.99	five +	p value		< 1 portion	1–4.99	five +	p value		< 1 portion	1–4.99	five +	p value	
Total count (n)	2752	1764	644			333	3545	1272			2884	2183	90			952	4052	156		
**Sex**				<0.001	Total (n)			0.353	Total (n)			<0.001	Total (n)			0.17	Total (n)
Male	51.1	32.2	16.7		2178	6.4	69.9	23.7		2175	59.9	38.1	2		2177	18.1	78.4	3.5		2178
Female	55	35.6	9.4		2982	6.5	68.1	25.4		2975	53	45.4	1.6		2980	18.7	78.6	2.6		2982
**Age**				<0.001					<0.001					<0.001					<0.001	
16–24	26.2	41.9	31.9		1004	10.8	78.5	10.7		1002	76.3	23.1	0.6		1004	30.8	68.2	1		1004
25–34	43.5	38.5	18		715	6	70.1	23.9		712	60.8	38	1.1		715	18.2	78	3.8		715
35–44	52.2	37.3	10.5		810	5.4	70.4	24.2		810	57.8	41.1	1.1		808	15.8	80.9	3.3		810
45–54	61.3	32.3	6.5		896	5.6	66.9	27.5		895	51.3	46.2	2.5		896	14.5	81.5	4		896
55–64	68	28	4		706	4.1	61.2	34.7		704	42.4	54.9	2.7		705	12.9	83	4.1		706
65+	70.6	27.1	2.3		1029	5.7	64.2	30.1		1027	44.4	53.1	2.5		1029	15.9	81.4	2.6		1029
**Education**				<0.001					<0.001					<0.001					<0.001	
Degree or higher	57.7	34.1	8.2		1123	1.8	57.8	40.4		1123	38.2	59.4	2.3		1122	8.1	85.2	6.7		1123
Below degree level	52.8	34.3	12.9		2309	5.5	71.1	23.4		2302	55.7	42.6	1.7		2307	17.1	80.5	2.4		2309
No qualifications	67.3	27.2	5.5		1031	11.2	70.7	18.2		1030	64.1	34	1.8		1031	24.6	73.7	1.6		1031
Full time student	27.4	44.5	28.1		697	10.4	76.4	13.2		695	73	26.3	0.7		697	30.6	68.3	1.1		697
**Housing tenure**				<0.001					<0.001					<0.001					<0.001	
Owned	54.7	35.2	10.1		3443	4.4	66.7	28.8		3439	49.1	48.8	2.1		3440	14.5	82.4	3.1		3443
Social Rent	52.5	30.3	17.2		917	13.1	75	11.9		916	73.8	25.1	1.1		917	31	67.6	1.4		917
Private rent	48.5	34.4	17.1		800	7.5	70.8	21.6		795	64.6	34.5	0.9		800	21.1	74.4	4.5		800
**Martial status**				<0.001					<0.001					<0.001					<0.001	
Single	38.6	38	23.3		1964	9.3	72.6	18.1		1961	67.9	30.9	1.2		1964	25.8	71.5	2.7		1964
Married	60.7	33.5	5.7		2093	3.4	65.3	31.3		2090	45.8	52.1	2.2		2091	11.2	84.9	3.9		2093
Separated/divorced/widowed	65.5	28.6	6		1103	7.2	68.9		23.9		1099	53.9	44.2	1.9		1102	19.1	79	1.9		1103

P values for Chi-square tests shown.

The results of the chi-square test for significance show the potential predictor variables (age, sex, marital status, tenure and educational qualifications) were all strongly correlated with diet outcomes with the exception of sex and fruit and vegetable portions and vegetable portions alone. All significant associations were confirmed with the logistic regression models. On the basis of these observed relationships, the SAE models for total fruit and vegetable portions and the model for vegetable portions only were created using age, tenure and martial status as the predictors. The model estimating the portions of fruit used age, tenure, marital status, and sex to predict this behaviour. The final model, estimating SSB consumption, used age, sex, martial status, and educational qualification.

The results in Table 2 summarise the least healthy dietary behaviour categories including high SSB consumption (more than one 330ml portion/day), lower fruit and vegetable consumption (less than one portion of fruit or one portion of vegetables, or less than one combined fruit, vegetables and 100% fruit juice portion/day).

**Table 2:** Mean prevalence of consumption for each diet variable by MSOA.

	Mean (%)	Minimum (%)	Maximum (%)	Std. Error	Std. Dev
>1 portion of fruit	54.25	44.15	76.10	0.06	5.24
< 1 portion of veg	17.32	12.78	30.74	0.04	2.93
< 1 portion of F&V	6.85	4.34	14.67	0.02	1.61
330 + ml SSB	11.47	5.69	30.51	0.03	2.63

The mean prevalence of adults consuming less than one portion of fruit, vegetables or 100% fruit juice is low overall, at 6.9% (range 4.3–14.7) of the population age 16+ years living in an MSOA. Comparing low levels of consumption of fruit or vegetables, estimates based on the NDNS dataset predict that fewer adults eat, on average, less than one portion of vegetables (17.3%, range 12.8–30.7) compared to fruit (54.3% [range 44.1–76.1]) per day. On average, 11.5% of the adult population in an MSOA are high SBB consumers (at least 330ml/day [range 5.7–30.5]). There is greater variation in the three single component variables (fruit, veg or SSB only) compared to the combined fruit and vegetable consumption (Table 2).

**Fig 2:**
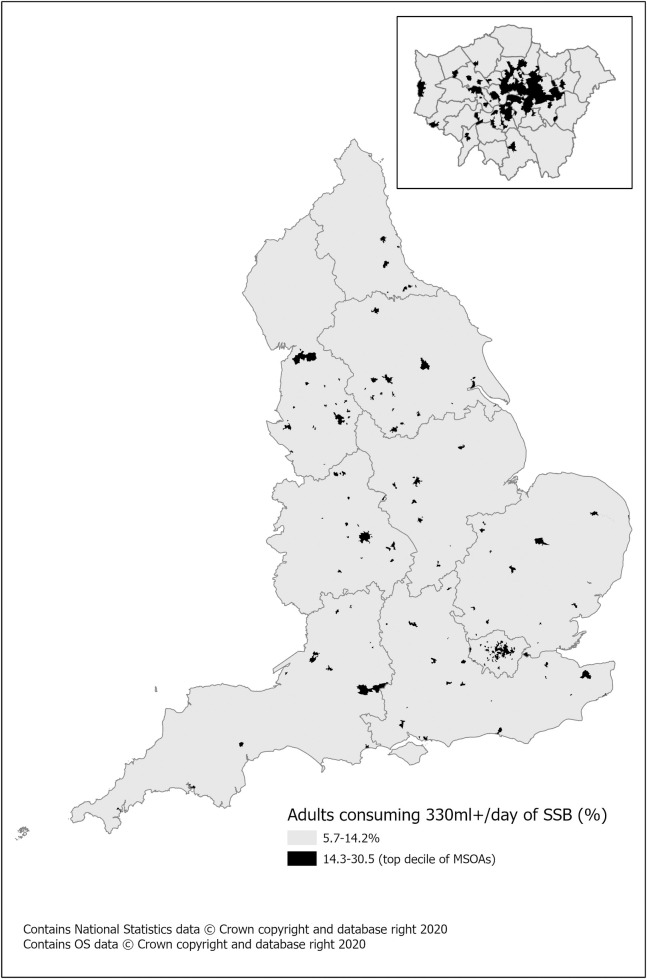
Estimated prevalence consuming more than 330ml SSB per day. This represents prevalence in adults by MSOA (2018 population) with inset of London.

The results for each of these estimated dietary variables were mapped to MOSAs in deciles with the results showing a distinct geography of higher SSB consumption, with greater prevalence (14.3–30.5% of the adult population drinking more than 330m/day) in areas within London, notably Tower Hamlets (Fig 2) [50]. Several areas along the south coast (Southampton, Portsmouth, Bristol), the midlands (Birmingham, Nottingham) and North (Leeds, Bradford, Liverpool, Manchester) also have relatively high prevalence of higher SSB consumption. All point estimates are available in S1 Table.

**Fig 3:**
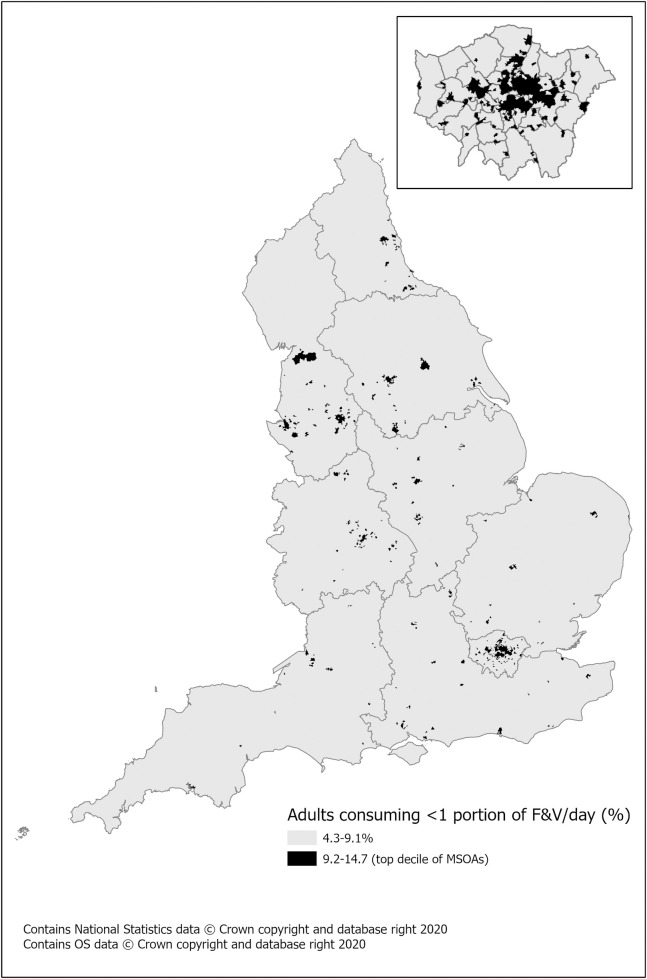
Estimated prevalence of consuming less than 1 portion of ‘five a day’. This measure includes 100% juice for adults by MSOA (2018 population) with inset of London.

The estimated prevalence of adults eating less than one portion of fruit, vegetables, or 100% fruit juice each day varies compared to the pattern of higher SSB consumption (Fig 3). Notably, northern cities (Leeds, Liverpool, Manchester) and some boroughs of London (Camden, Haringey, Hackney, Tower Hamlets, Newham) have higher rates of the poorest level of fruit and vegetable consumption as well as Birmingham and Southampton. This is defined as MSOAs where 9.2–14.7% of the adult population consume less than a portion of fruit or veg/day, which is top decile of MSOAs for this measure of diet quality.

**Fig 4:**
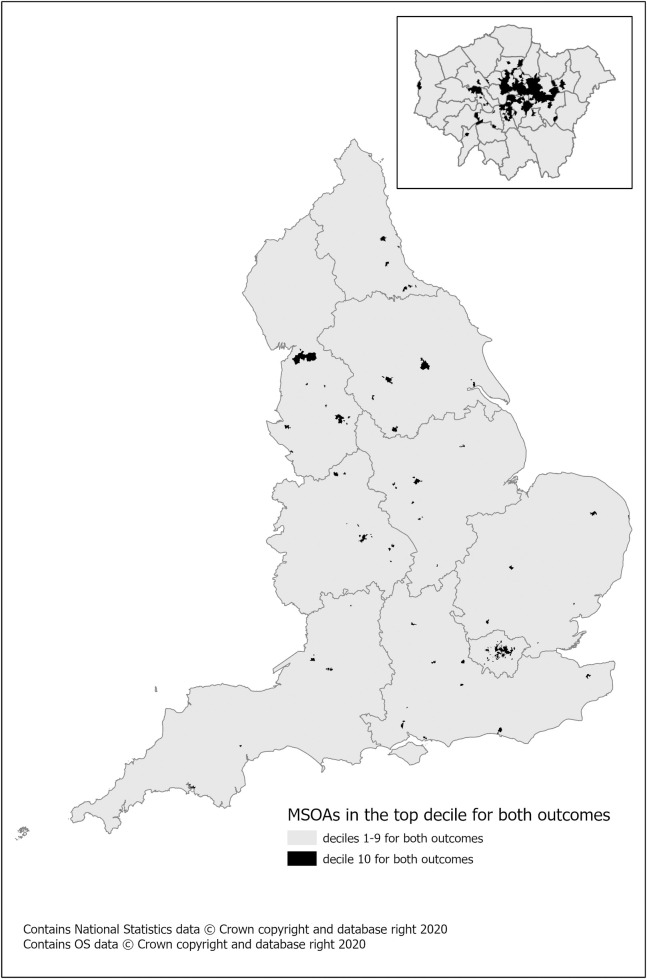
Combined poor diet prevalence. Deciles of highest SSB consumption and highest percentage of the population eating less than one portion of fruit and vegetable a day) by MSOA (2018 population) with inset of London.

A subset of areas showed low fruit and vegetable consumption predicted alongside higher prevalence of greater SSB consumption. There are 356 MSOAs (5.2% of England) where more than 9.2% of the population are estimated to consume less than a portion of fruit, vegetables or juice a day and in each of these MSOAs more than 14.3% of the population are estimated to drink >300ml of SSB a day (Fig 4). These MSOAs include areas from Birmingham, Leeds, and Manchester and Bristol. The areas included in this combined higher risk group are predominantly urban, as clearly illustrated in the London inset map (Islington, Newham, Hackney and Tower Hamlets).

For the two key sets of diet estimates, the MSOAs identified as being in the top decile of prevalence for poor diet outcomes were significantly different from the other 90% of areas using the Mann-Whitney U test (p<0.001).

In order to identify local authorities where the less desirable health outcomes are most prevalent and clustered spatially, we used Local Moran’s I test for spatial autocorrelation. The results from this analysis identify more areas where MSOAs with less desirable diet outcomes are more prevalent, however, as the test here is for spatial autocorrelation, there are many more areas identified in clusters of high prevalence of the key diet outcomes that are surrounded by similar areas (1,569 MOSAs for low fruit and vegetable consumption, 2,038 MOSAs for high SSB consumption). These clusters will help to identify local authorities where prioritisation of diet interventions could be enacted, while the decile distribution is more appropriate for within-local authority prioritisation. Maps of the results from the Moran’s I are in S1 Fig.

The variables that were used to estimate the dietary behaviours such as age and sex are also estimated in the model to provide an indication of internal model goodness-of-fit by comparing to the known population distribution from the ONS mid-year estimates. The mean TAE (mTAE) for each variable was low. For SSB the mTAE were 2.1 E-15, 0.00067, 0.009 and 0.014 with age being the variable with the highest average percent error. In the total portion variables, the mTAE values were 2.01 E-15, 0.0001 and 0.002 with age showing the most error. The values were similarly very low for portions of fruit and portions of vegetables. This internal error is well within the suggested limits for internal goodness-of-fit, where error is less than 20% in at least 80% of the study areas [51].

Data on adult fruit and vegetable consumption at the 2011 local authority district level (n = 326, with an average of 21 MSOAs in each) was available from the Active Lives Survey 2016/17 [52], however, analysis of this survey data showed that respondents were far more likely to have five portions of fruit and vegetables a day (58.1% of those age 16+ years) than respondents from the NDNS (24.7%). This disparity in descriptive statistics between the NDNS used to model the outcomes and data collected in the Active Lives survey would incorrectly indicate that there was a high proportion of error in the estimated data based on NDNS respondents. The Active Lives Survey asks only the screener question about the portions of fruit and vegetables which is prone to error when a more in-depth dietary assessment method is used such as the four-day food diary in NDSNS. This may explain the disparity between the reported prevalence of people achieving five a day between the two surveys.

**Fig 5:**
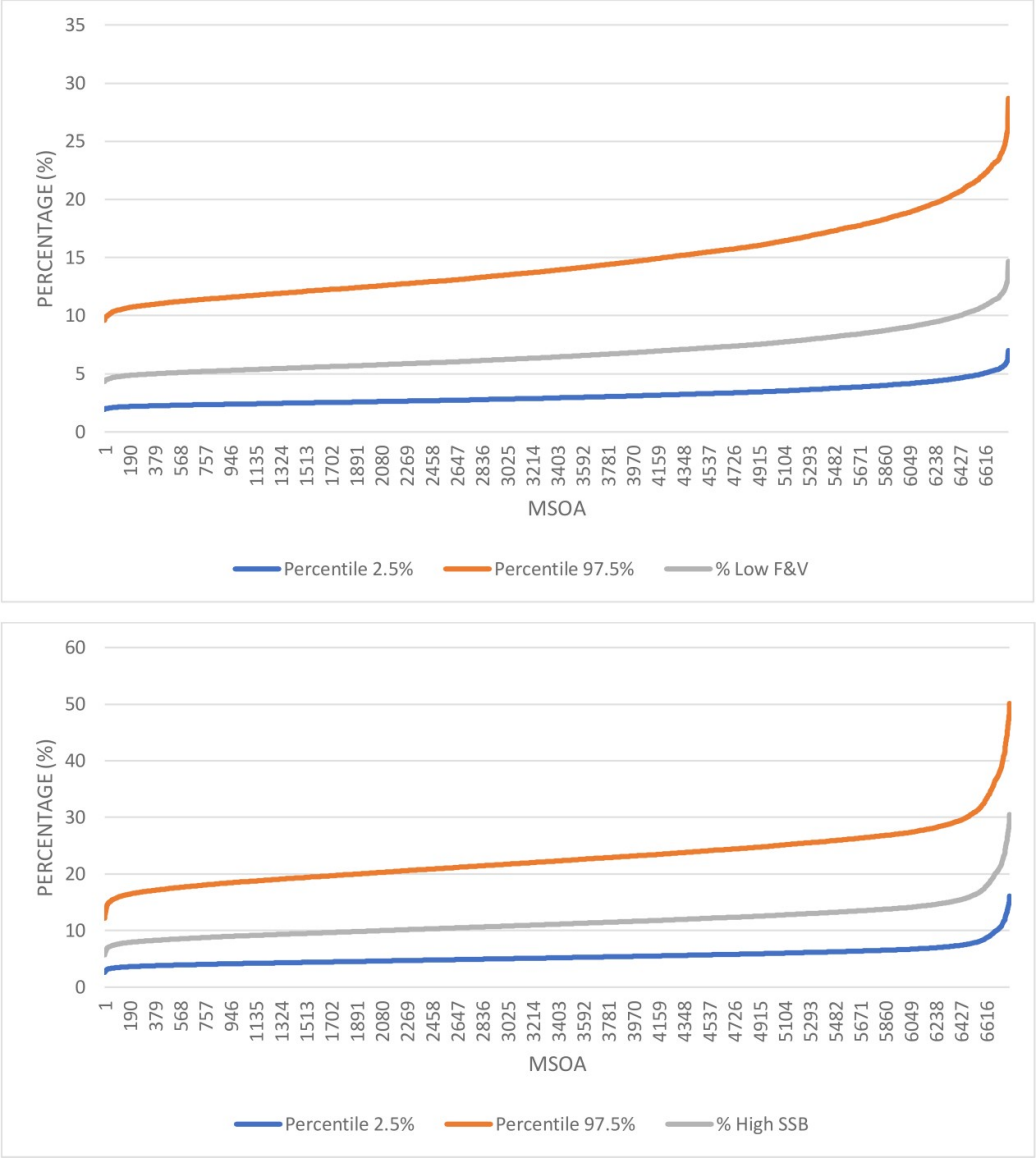
Credible intervals. Credible intervals around a sample of the estimates of SSB and overall fruit and vegetable consumption.

The credible intervals reflect the uncertainty in the estimated values. They are wider for the outcomes where the original regression model results showed a weaker relationship between the predictor variables and the diet outcome. An example of this is the relatively tight credible intervals for low levels of fruit and vegetable consumption (less than one portion/day). On average, the estimated prevalence of adults age 16+ who did not have at least on portion a day was 6.9% with an average credible interval of 3.1% - 14.6%. In contrast, the average value for drinking at least 330ml of SSB/day was 11.5% with a wider credible interval of 5.4–22.8% (average values). The credible intervals are not symmetrical around the point estimates as they have a lower limit of zero, so the upper limit will be greater from the point estimate to reflect this value constraint (Fig 5).

## Discussion

The estimated diet behaviours in the adult population show that in a small number of MSOAs, two poor diet behaviours (more than 330ml/SSB/day or less than portion of fruit, vegetable or 100% fruit juice/day) are more prevalent. Relatively high SSB consumption (at least 330 ml/day) was more common than estimated prevalence of low daily fruit and vegetable intake (< 1 portion of fruit, vegetables of 100% juice/day). These areas could benefit from closer consideration by local public health teams for area-based interventions to promote healthier diet behaviours.

Results of the SSB model highlight inner London and Northern areas where there is a higher proportion of more socially and materially deprived populations. Here, more of the adult population are likely to experience a greater impact of the sugar tax on the cost of their beverage consumption. The data from this study show high (>330 ml/day) SSB consumption is more common in areas with greater numbers of younger adults as seen in earlier research [4] and higher proportions of full-time students.

The impact of the sugar tax, a regressive approach to taxation would usually be argued to affect the lowest income groups most. Indeed, the data from the NDNS data confirms higher prevalence of >330ml consumption in the lowest social group as classified by NSSEC. Although the sugar tax has promoted some reformulation of products to reduce sugar, in some situations the higher costs for producers is passed on to consumers. The results of this study suggest that areas of inner London, and other cities including Leeds, Birmingham and Liverpool would experience the greatest impact. These results agree with the geographic pattern identified by Collins et al. [36] when reductions of kcal/day following a 20% duty on SSB were estimated at the LAD level. One further option to reduce adult SSB consumption is to target retailers in the areas where high consumption is estimated to be greatest to promote non-SSB alternatives.

Greater prevalence of low consumption of fruit and vegetables show a similar geography to high SSB with overlapping areas in London, however, there are parts of Liverpool, Birmingham and Leeds where a higher proportion of the adult population are estimated to consume <1 portion of fruit, vegetables or fruit juice each day. There are 326 MSOAs identified where the model estimates predict both relatively high SSB consumption and low fruit and vegetable consumption. Most of these areas are located in the northern part of the country and inner London, which is supported by previous research showing greater inequalities in health between the North and South of England [53, 54]. Stronger local interventions to actively support more fruit and vegetable consumption, such as local voucher schemes or targeted redistribution of fresh produce to these neighbourhoods through existing organisation (FareShare) may have the greatest benefit for addressing diet-related health inequalities in these populations.

These data will be particularly helpful for Local Authorities who hold responsibility for localised public health campaigns [24], monitoring trends over time or proposing interventions. In combination with enhanced provision of fruit and vegetable vouchers or redistribution in areas identified by the modelling, local interventions may be prioritised to localities where the impact can be greatest. This illustrates one strength of small-area estimation in the absence of comprehensive local surveys, as they allow public health teams in local authorities to identify priorities for local population health inequalities. For this reason, Public Health England regularly commissions small-area estimation models of behaviours such as smoking and outcomes like obesity or depression; this model is an extension to include additional behaviours which would benefit from focused interventions. The results of this model will provide health strategists with data to support local intervention development or help evaluate the potential effectiveness or best location for interventions. Previous scenario modelling using spatial microsimulation provided insight for the optimal locations of smoking cessation services [18] and a similar approach may be applied for diet interventions as described above. A notable benefit of the model presented here is the use of credible intervals around prevalence estimates to demonstrate uncertainty. Only recently have these intervals been added to spatial microsimulation outputs, the inclusion of credible intervals here will allow for more confident use of these data.

The estimates for diet are built using the demographic profiles of respondents to the NDNS survey and attributing the observed diet behaviours to local area populations based on the known population characteristics from the Census and ONS updates. One limitation to this method is the local population data is not as current as we would prefer. In addition, this model assumes that all people replicate the behaviours of respondents to the NDNS. As the NDNS data are used for annual updates of population-level diet behaviours, this is a consistent assumption and the same SAE method has been successful in modelling other health outcomes and behaviours such as obesity [15] common mental disorders [17] and smoking [42].

The width of credible intervals reflect the relatively small sample size of respondents from the NDNS, which was the most appropriate dataset to capture a range of diet outcomes; the Health Survey for England (average annual sample of approximately 10,000) stopped consistently collecting data on dietary behaviours beyond a screener to collect consumption of fruit and vegetables in 2012 [38]. In addition, NDNS collects data using a four-day food diary which is more reliable than a screener based on the last 24 hours of consumption as seen in Health Survey for England and the Active Live Survey [55].

## Conclusion

The results of this study provide an indication of geographic areas where diet-related policy interventions such as voucher schemes or redistribution services may have the greatest impact; urban areas in the north of England and some southern coastal cities should be prioritised for dietary improvements. Population-based policy interventions such as taxes to reduce SSB consumption can be evaluated using these local estimates as a baseline. There is also capacity to use the results for optimally locating targeted interventions such as increased Healthy Start or similar voucher values in selected areas. This modelling is a valuable tool which may be easily adopted by local authorities as they maintain responsibility for prioritising health campaigns to reduce health inequalities in their populations, and further validated as more data becomes available. The methods used in this study can be replicated in any country with national survey data on dietary variables.

## Supporting information

S1 Fig Local Moran’s I clusters of diet behaviours. (a) Clusters of high SSB consumption and (b) low fruit and vegetable consumption.
(TIF)

S1 Table Point estimates of diet behaviours in MSOAs. Prevalence of diet behaviours in adults using 2018 population estimates.
(CSV)
