# Supplementary figures and images for: Adult diet in England: Where is more support needed to achieve dietary recommendations?
Source: PLoS One. 2021 Jun 23;16(6):e0252877. doi: 10.1371/journal.pone.0252877 (PMC8221484; doi:10.1371/journal.pone.0252877)

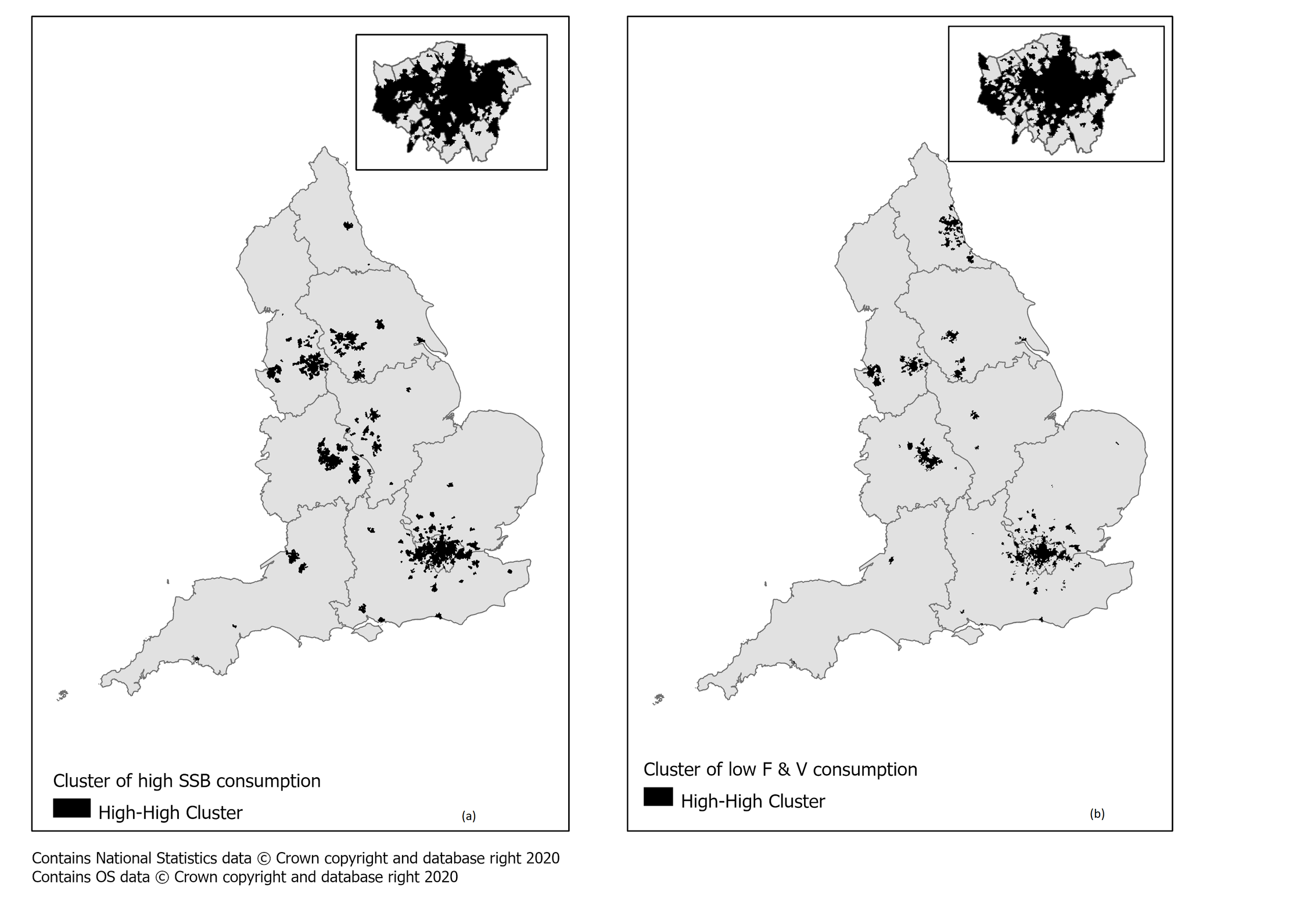

Supplement: S1 Fig — (a) Clusters of high SSB consumption and (b) low fruit and vegetable consumption. (TIF) [file pone.0252877.s001.tif]
